# An imperative need to change pharmacology curriculum: A pilot survey

**DOI:** 10.4103/0253-7613.71901

**Published:** 2010-12

**Authors:** K. Vasundara, P. Kanchan, H.P. Pundarikaksha, K. Girish, S. Prassana, R. Jyothi

**Affiliations:** Department of Pharmacology, Kempegowda Institute of Medical Sciences, Bangalore 560 070, India

Sir,

Pharmacology being both a basic and applied science forms the backbone of rational therapeutics. The primary objective of teaching pharmacology is to enable undergraduate medical students to take rational therapeutic decisions in clinical practice. However, this objective is not adequately met by the prevailing curricula.[1] The subject is taught with high factual information rather than therapeutic skills. Hence, a pilot survey was carried out at our teaching hospital to assess the clinical application of the pharmacology knowledge in patient care. The following questionnaire was distributed to interns. A 4-point Likert scale ranging from strongly disagree to strongly agree was used.

1. Relevance of basic experimental pharmacology (experiments on isolated frog tissues and rabbits’ eyes) knowledge in patient care.
2. Relevance of dispensing pharmacy exercise knowledge (preparing and dispensing of ointments, liniments, pastes, etc.) in patient care.
3. Do you feel bedside case study (a real clinical problem) in pharmacology practical would have been more relevant in patient care?
4. Necessity for integrating clinical pharmacology with clinical subjects in final MBBS phase-3.

**Figure 1:**
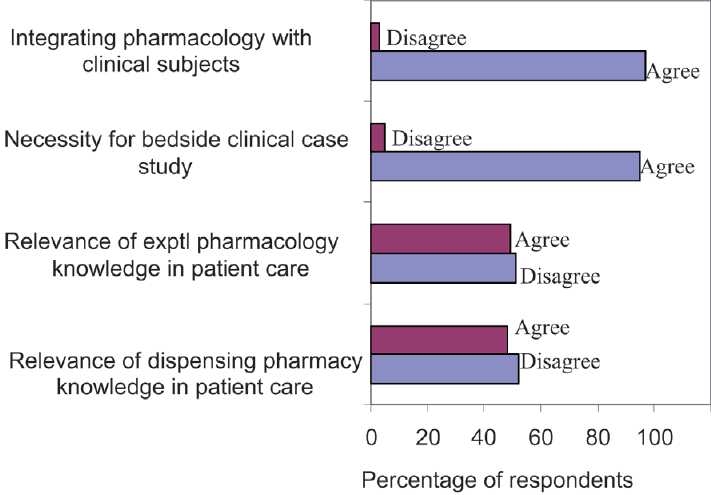
Comparison of responses (%) of participants to questionnaire (n = 74).

The data were compiled and analysed. Out of 82 interns, 74 responded completely to the questionnaire. The survey indicated that relevance of experimental pharmacology and dispensing pharmacy knowledge in patient care was poor [Figure 1]. Our study substantiated the view that practical curricula are “outdated and obsolete, and has failed to achieve the objective for which they were instituted”.[2]

Majority of the interns (95%) felt necessity for bedside clinical case study and the necessity of integrating pharmacology teaching with clinical subjects in MBBS phase-3, i.e. context learning–gaining of knowledge and skills simultaneously (97%). This corroborates with studies done in other countries where context learning has been found to be more successful and effective than sequential learning where in learning and applications of knowledge are separated.[3,4] The clinical postings of undergraduates emphasize on teaching diagnosis of the diseases. The skills required for therapeutic reasoning and prescribing are not addressed and taught in a structured way.[4,5] On the other hand, interns are expected to prescribe drugs from the first day of clinical work and may not feel confident when confronted to take decisions independently.

Thus, our study upholds the view that there is an imperative need to implement radical changes in the teaching curricula of pharmacology which should be in consonance with patient care for the doctors of tomorrow to render better health service.
